# Comparing the EQ-5D-3 L and EQ-5D-5 L: studying measurement and scores in Indonesian type 2 diabetes mellitus patients

**DOI:** 10.1186/s12955-020-1282-y

**Published:** 2020-02-07

**Authors:** Bustanul Arifin, Fredrick Dermawan Purba, Hendra Herman, John M. F. Adam, Jarir Atthobari, Catharina C. M. Schuiling-Veninga, Paul F. M. Krabbe, Maarten J. Postma

**Affiliations:** 1 Department of Health Sciences, University of Groningen, University Medical Center Groningen, University of Groningen, Hanzeplein 1, Groningen, 9700 RB The Netherlands; 2 Faculty of Pharmacy, Hasanuddin University, Makassar, Indonesia; 3 Institute of Science in Healthy Ageing & healthcaRE (SHARE), University Medical Center Groningen (UMCG), University of Groningen, Groningen, The Netherlands; 4 Disease Prevention and Control Division, Banggai Laut Regency Health, Population Control and Family Planning Service, Central Sulawesi, Indonesia (Bidang Pencegahan dan Pengendalian Penyakit, Dinas Kesehatan, Pengendalian Penduduk & Keluarga Berencana, Pemerintah Daerah Kabupaten Banggai Laut, Jl. Jogugu Zakaria No. 1, Banggai, Sulawesi Tengah, Indonesia; 5 Unit of Pharmacotherapy, Epidemiology & Economics (PTE2), Department of Pharmacy, University of Groningen, Groningen, The Netherlands; 6 Department of Developmental Psychology, Faculty of Psychology, Universitas Padjadjaran, Jatinangor, Indonesia; 7 Faculty of Pharmacy, Universitas Muslim Indonesia, Makassar, Sulawesi Selatan Indonesia; 8 Pharmacy Department, Ibnu Sina Hospital, Makassar, Sulawesi Selatan Indonesia; 9 Division of Endocrinology and Metabolism, Department of Internal Medicine Faculty of Medicine Hasanuddin University Makassar, Makassar, Indonesia; 10 Department of Pharmacology and Therapy, Faculty of Medicine, Public Health and Nursing, Universitas Gadjah Mada, Yogyakarta, Indonesia; 11 Clinical Epidemiology and Biostatsitic Unit, Faculty of Medicine, Public Health and Nursing, Universitas Gadjah Mada, Yogyakarta, Indonesia; 12 Department of Epidemiology, University Medical Center Groningen, University of Groningen, PO Box 30.001, 9700 RB, Groningen, The Netherlands; 13 Department of Economics, Econometrics & Finance, Faculty of Economics & Business, University of Groningen, Groningen, The Netherlands; 14 Department of Pharmacology and Therapy, Faculty of Medicine, Universitas Airlangga, Surabaya, Indonesia

## Abstract

**Background:**

The EuroQoL five-dimensional instrument (EQ-5D) is the favoured preference-based instrument to measure health-related quality of life (HRQoL) in several countries. Two versions of the EQ-5D are available: the 3-level version (EQ-5D-3 L) and the 5-level version (EQ-5D-5 L). This study aims to compare specific measurement properties and scoring of the EQ-5D-3 L (3 L) and EQ-5D-5 L (5 L) in Indonesian type 2 diabetes mellitus (T2DM) outpatients.

**Methods:**

A survey was conducted in a hospital and two primary healthcare centres on Sulawesi Island. Participants were asked to complete the two versions of the EQ-5D instruments. The 3 L and 5 L were compared in terms of distribution and ceiling, discriminative power and test-retest reliability. To determine the consistency of the participants’ answers, we checked the redistribution pattern, i.e., the consistency of a participant’s scores in both versions.

**Results:**

A total of 198 T2DM outpatients (mean age 59.90 ± 11.06) completed the 3 L and 5 L surveys. A total of 46 health states for 3 L and 90 health states for 5 L were reported. The ‘11121’ health state was reported most often: 17% in the 3 L and 13% in the 5 L. The results suggested a lower ceiling effect for 5 L (11%) than for 3 L (15%). Regarding redistribution, only 6.1% of responses were found to be inconsistent in this study. The 5 L had higher discriminative power than the 3 L version. Reliability as reflected by the index score was 0.64 for 3 L and 0.74 for 5 L. Pain/discomfort was the dimension mostly affected, whereas the self-care dimension was the least affected.

**Conclusions:**

This study suggests that the 5 L-version of the EQ-5D instrument performs better than the 3 L-version in T2DM outpatients in Indonesia, regarding measurement and scoring properties. As such, our study supports the use of the 5 L as the preferred health-related quality of life measurement tool.

We did not do a trial but this study was approved by the Medical Ethics Committee of Universitas Gadjah Mada Yogyakarta, Indonesia (document number KE/FK/1188/EC, 12 November 2014, amended 16 March 2015).

## Introduction

In 2011, the number of people suffering from diabetes mellitus (DM) in the world was reported at 366 million [1]. Based on the latest data in 2017, this number has increased by almost 20% to reach 450 million [2]. Worldwide, 90% of these suffer from type 2 diabetes mellitus (T2DM) [3]. In Indonesia, in the same period mentioned, the number of people with T2DM even increased by 30%, i.e., from 7.3 million to 10.3 million [1, 2]. In this respect, the Indonesian Ministry of Health also reported that the national prevalence of T2DM in Indonesia had almost doubled from 1.1% in 2007 to 2.1% in 2013 [4]. Furthermore, the Ministry of Health’s report stated that of the 34 provinces in Indonesia, 15 provinces had a higher prevalence of T2DM patients than the national average, inclusive Sulawesi island [4]. Notably, the prevalence of T2DM amounts to 3.7% in Central Sulawesi province, 3.6% in North Sulawesi and 3.4% in South Sulawesi [4]. The continued increase in the prevalence of T2DM patients in Indonesia requires serious attention, especially concerning control of T2DM costs and patients’ health status and cost-effectiveness of interventions. In this respect, adequate measurement of health-related quality of life (HRQoL) reflects a core issue.

The EuroQoL five-dimensional instrument (EQ-5D) is the recommended preference-based instrument to measure HRQoL in several countries [5, 6]. HRQoL is measured by this instrument in such a way that it generates a single index score or utility. This instrument consists of five items covering five health-state dimensions (mobility, self-care, usual activities, pain/discomfort, and anxiety/depression), with each item originally having three levels of severity (EQ-5D-3 L) [7]. In 2011, the EuroQol Group expanded the number of severity levels for each dimension to five (EQ-5D-5 L) [8]. Both the EQ-5D-3 L (3 L) and EQ-5D-5 L (5 L) versions have been used in several studies, covering both clinical and methodological assessments [8–10].

Several comparative studies of the 3 L and 5 L versions of EQ-5D have been conducted in the countries neighbouring Indonesia, notably Singapore and Thailand. Both studies reported that 5 L is the preferable version for T2DM patients considering its greater discriminative power and patients’ preferences [11, 12]. Considering the 5 L and 3 L versions, it is noted that both versions have been used in several studies in Indonesia, already, but a structured, integrative and direct comparison is still lacking [13–16], however a structured integrative comparison is still missing, motivating the conduct of our study. Whereas such comparisons would be available for other countries, sociodemographic characteristics and cultural differences between Indonesia and other countries might differ potentially resulting in varying findings measurement properties of the two EQ-5D versions. Therefore, this study aims to directly compare specific measurement properties and scorings of the 3 L and 5 L versions in Indonesian type 2 diabetes mellitus (T2DM) outpatients.

## Materials and methods

### Study design

A cross-sectional study was conducted from July 2016 to April 2017. A secondary care setting in South Sulawesi and two primary care settings in Central Sulawesi were included. In particular, these were Jaury Academic Hospital in Makassar and the Puskesmas/primary healthcare centers (PHCs) in Simpong and Kampung Baru in Luwuk Banggai, respectively. This study was approved by the Medical Ethics Committee of Universitas Gadjah Mada Yogyakarta, Indonesia (document number KE/FK/1188/EC, 12 November 2014, amended 16 March 2015).

### Participants

Participants were T2DM outpatients with a minimum age of 18 years. The participants were informed of the study objectives and study procedure. The researcher or research assistants obtained signed informed consent forms from the participants. For the participants with disabilities or difficulties in reading, consent was based on confirmation from their caregiver who accompanied them during treatment at a health facility. The caregiver played a role in providing support to the participants as they filled in the instruments. It is important to note that all decisions on the exact health states chosen originated from the participants. In this study, all participants were treated by a consulting resident internal medicine who gave his/her consent to the data collection during the participant’s T2DM consultation (in primary and secondary care).

### Instruments

EQ-5D 3 L and 5 L consist of two parts: the EQ-5D descriptive system classification and the EQ visual analogue scale (EQ-VAS). The EQ-5D descriptive system comprises five items on its HRQoL dimensions: mobility, self-care, usual activities, pain/discomfort, and anxiety/depression. Each dimension in the 3 L version [10] is completed with three response options: no problem, some problems, and confined to bed/unable/extreme problems, yielding a possible 243 (3^5^) unique health states. A single digit expresses the level selected for that specific dimension. Therefore, the five-digit number for five dimensions describes a specific health state. For example, ‘11111’ indicates ‘no problems on any of the five dimensions’, while ‘23231’ indicates ‘some problems walking, unable to wash or dress, some problems with performing usual activities, extreme pain/discomfort, and no anxiety/depression’. The 5 L [8] has five scale options to choose from: no problem, slight problems, moderate problems, severe problems, and extreme problems/unable. The 5 L instrument yields 3125 (5^5^) unique health states. For example, ‘12345’ indicates ‘no problems walking, slight problems washing or dressing, moderate problems doing usual activities, severe pain/discomfort and extreme anxiety/depression’. The EQ-VAS presents the participants’ self-rated health on a scale of 0 (worst imaginable health) to 100 (best imaginable health). The time frame for the EQ-VAS is ‘today’, meaning that participants were asked to describe their health state during the day they were interviewed. We used the 3 L and 5 L Bahasa Indonesia versions of the EQ-5D, produced by the EuroQol Group using a standardized translation protocol [17] and having been proved as valid and reliable questionnaires in Indonesian patient groups [13–16].

### Data collection procedure and data sources

After introducing the researchers and explaining the purpose of the study, a brief description to the participants was provided on how to use the EQ-5D instruments. An explanation of the concept of HRQoL as an aid on how they should describe their health state was presented. The participants were given the opportunity to ask questions throughout the data collection process. For EQ-VAS, we asked the participants to describe their health state and provide the most appropriate score to define their health state. Three research assistants were hired to collect the data. As a sequence, participants first classified their health state on the 5 L items, then provided their data (sociodemographic and clinical conditions), followed by the 3 L.

According to socio-demographic data (gender, age, T2DM duration, occupation, level of education, and dependence on a caregiver) were obtained from self-reporting. In this study, participants were classified into two age categories based on the retirement age of Indonesian people (56 years): productive age (below 56 years) and retirement age (56 years and above). As for employment status, participants were defined as in active employment when they were still actively working, and unemployed if they reported not having a job. Those whose main responsibilities were for their family members and household chores were classified as housewives.

Data on the clinical conditions, such as the type of therapy, T2DM-related complications, and comorbidities were obtained from treating physicians. Self-reported data from participants was used in the cases data could not be collected through the treating physicians. In this study, participants were defined as having comorbidities if they suffered from other diseases, such as asthma, gastritis and gout problems. Participants were defined as having complication and comorbidities if they suffered from other diseases and T2DM complications; for example, a participant with comorbid cancer and hypertension as a complication of diabetes.

### Test-retest reliability

Test-retest reliability was analyzed using sequential measurements. Participants involved in this phase were those who visited the specific health facility twice. The time interval between the two measurement times was four weeks as the participants were scheduled to meet their consulting resident internal medicine each month. Notably, an additional question was asked before the participants completed the instruments the second round: ‘Has there been any major change in your health state between the first time you completed the instruments last month and today? For example, have you been hospitalised, had an accident, experienced a natural disaster or have been bereaved’? Participants who answered ‘yes’ were excluded from the final sample.

### Analyses

For self-reported health state profiles obtained from the two versions of EQ-5D, we calculated the percentage of participants who responded to each level of each dimension. To determine the consistency of the participants’ answers, we checked the redistribution pattern, i.e., the consistency of individual participants’ scores in both versions. A consistent response pair was defined as a 3 L response which is at most one level away from the 5 L response (e.g., a participant chose level 1 in 3 L and chose level 2 in 5 L). When the 5 L level was more than 1 level away from the 3 L level (e.g., a participant chose level 1 in 3 L and chose level 3 in 5), this was labelled inconsistent [11]. Next, we converted their scores on 3 L to 5 L as follows: 1 in 3 L equals 1 in 5 L, 2 in 3 L equals 3 in 5 L, and 3 in 3 L equals 5 in 5 L [12]. The ceiling effect was defined as the proportion of participants who reported not having problems in any of the five EQ-5D dimensions (health state ‘11111’) for both 3 L and 5 L. This statistic is often used to assess the discriminatory power of health-state classification systems [18, 19]. As Indonesia only has the EQ-5D-5 L value set, not the 3 L [20], to obtain consistent 3 L and 5 L utility index scores, the UK 3 L and 5 L value sets [21, 22] were used.

The test-retest reliability of the dimension scores was assessed using the weighted kappa. We applied Landis JR & Koch GG standards [23] to determine the strength of agreement of the kappa values as follows: < 0.00 = poor, 0.00–0.20 = slight, 0.21–0.40 = fair, 0.41–0.60 = moderate, 0.61–0.80 = substantial, and 0.81–1.00 = almost perfect [20]. The test-retest reliability of the EQ-VAS and index scores were calculated using intra-class correlation coefficients (ICCs), two-way random effects and absolute agreements. The following reliability guideline was used for the strength of the ICC values: < 0.5 = poor, 0.5–0.75 = moderate, 0.75–0.90 = good and > 0.90 = excellent [24]. The discriminative power was calculated using the Shannon index (H′) and Shannon’s Evenness index (J’) [18, 19]. The Shannon index combines the absolute information content as expressed by the number of categories with the extent to which the information is evenly spread over these categories. On the other hand, the J’ expresses the relative information of a system or the evenness of the information distribution regardless of the number of categories. In case of an even distribution, when all levels are filled with the same frequency, J’ is equal to 1. Larger H′ and J’ values indicate more discriminatory performance. All the data were analysed using IBM SPSS Statistics for Windows version 23 (SPSS Inc., Cambridge, MA, USA), and statistical significance was set a priori at *p* < .05.

## Results

### Descriptive

A total of 198 participants were interviewed (Table 1). The average age of the participants was almost 60 years, with 58% being female, and 70% of female participants reported being housewives as their main activity. Regarding the clinical conditions, more than 70% of participants were being treated with oral antidiabetic therapy (OAD), both monotherapy and OAD combinations, and 52% of participants reported T2DM-related complications. Furthermore, participants had various comorbidities, such as asthma (*n* = 6), gastritis (*n* = 5), and gout (*n* = 3).

**Table 1:** Sociodemographic characteristics, clinical conditions and participants’ preferences

Variables	Overall (*n* = 198)
	n (%)
Sociodemographic characteristics	
Mean age (year) ± SD	59.90 ± 11.06
Age*	
Less than 56	70 (35)
More than 56	128 (65)
Sex	
Male	84 (43)
Female	114 (57)
Education level	
None	3 (2)
Primary school	33 (16)
Junior high school	42 (21)
Senior high school	83 (42)
University degree	37 (19)
Occupation	
Employed	64 (32)
Retired	53 (27)
Housewife	80 (41)
Caregiver	
No	125 (63)
Yes	73 (37)
Clinical conditions	
Type of therapy	
Diet or no OAD or insulin in the R/**	20 (10)
OAD (mono and combinations)	143 (72)
Insulin (mono and OAD combinations)	35 (12)
Complications and comorbidities	
None	74 (38)
Yes	103 (52)
Comorbidities^a^	14 (7)
Complications and comorbidities^b^	7 (3)
Types of complications	
No	74 (38)
Microvascular	18 (9)
Macrovascular	78 (40)
Micro & macrovascular	7 (3)
Number of T2DM complications	
No	74 (38)
One complication	76 (39)
Two or more	27 (13)

*We choose 56 years as the cut-off point because that is the pension age in Indonesia

^a^Participants were defined as having comorbidities if they suffered from other diseases (not T2DM complications)

^b^Participants were defined as having complication and comorbidities if they suffered from other diseases and T2DM complications

For test and re-test reliability, of the 198 participants who completed the first survey, 53 participants (62% female) completed the instruments twice. In this phase, only 12 participants had a university degree and most of the female participants were housewives (*n* = 20). Furthermore, of the almost 70% of participants treated with OADs, 40% reported T2DM without complications and 36% reported T2DM with at least one complication. There were no missing health state data.

### Scoring and ceiling

Participants usually reported no problems (level 1) on both 3 L and 5 L, except for the pain/discomfort dimension with only 25 and 20% of participants reporting no problems on 3 L and 5 L, respectively. Therefore, pain/discomfort was more often reported at other 3 L and 5 L levels compared to the other EQ-5D dimensions (Table 2).

**Table 2:** Self-reported health on the EQ-5D-3 L and EQ-5D-5 L descriptive system, and the EQ-VAS

	EQ-5D-3 L			EQ-5D-5 L				
Dimensions & VAS	No problems (%)	Some problems (%)	Unable/ Extremely problems (%)	No problems (%)	Slight problems (%)	Moderate problems (%)	Severe problems (%)	Unable/ Extremely problems (%)
Mobility	58.38	41.62	0.00	50.51	24.24	12.62	11.62	1.01
Self-care	82.23	16.75	1.02	78.28	12.63	5.05	3.03	1.01
Usual activities	67.51	28.43	4.06	63.64	18.18	7.58	7.07	3.54
Pain/ discomfort	25.38	59.90	14.72	19.70	40.91	18.18	17.17	4.04
Anxiety/ depression	46.70	44.67	8.63	43.43	33.84	12.63	8.00	2.02
Mean EQ-VAS (SD)	74.71 (20.13)			74.81 (19.70)				
25% percentile	60.00			60.00				
50% percentile	75.00			75.00				
75% percentile	90.00			90.00				

*VAS* Visual analogue scale

Regarding the ceiling effect, the 5 L version showed slightly fewer reports of absence of problems in all dimensions (‘11111’) compared to the 3 L version. The percentage of participants reporting the ‘11111’ health state decreased from 15% in the 3 L to 11% in the 5 L. Nevertheless, no statistically significant difference was found (*p*-value = .178). Self-care reached the highest ceiling (82% for the 3 L, 78% for the 5 L) while pain/discomfort showed the lowest ceiling (as mentioned above, 25% for the 3 L, 20% for the 5 L). The anxiety/depression dimension showed the smallest reduction in the ceiling (3% less), whereas the mobility dimension showed the largest reduction (7% reduction) when going from 3 L to 5 L. None of the ceiling reductions from 3 L to 5 L were statistically significant.

**Fig. 1:**
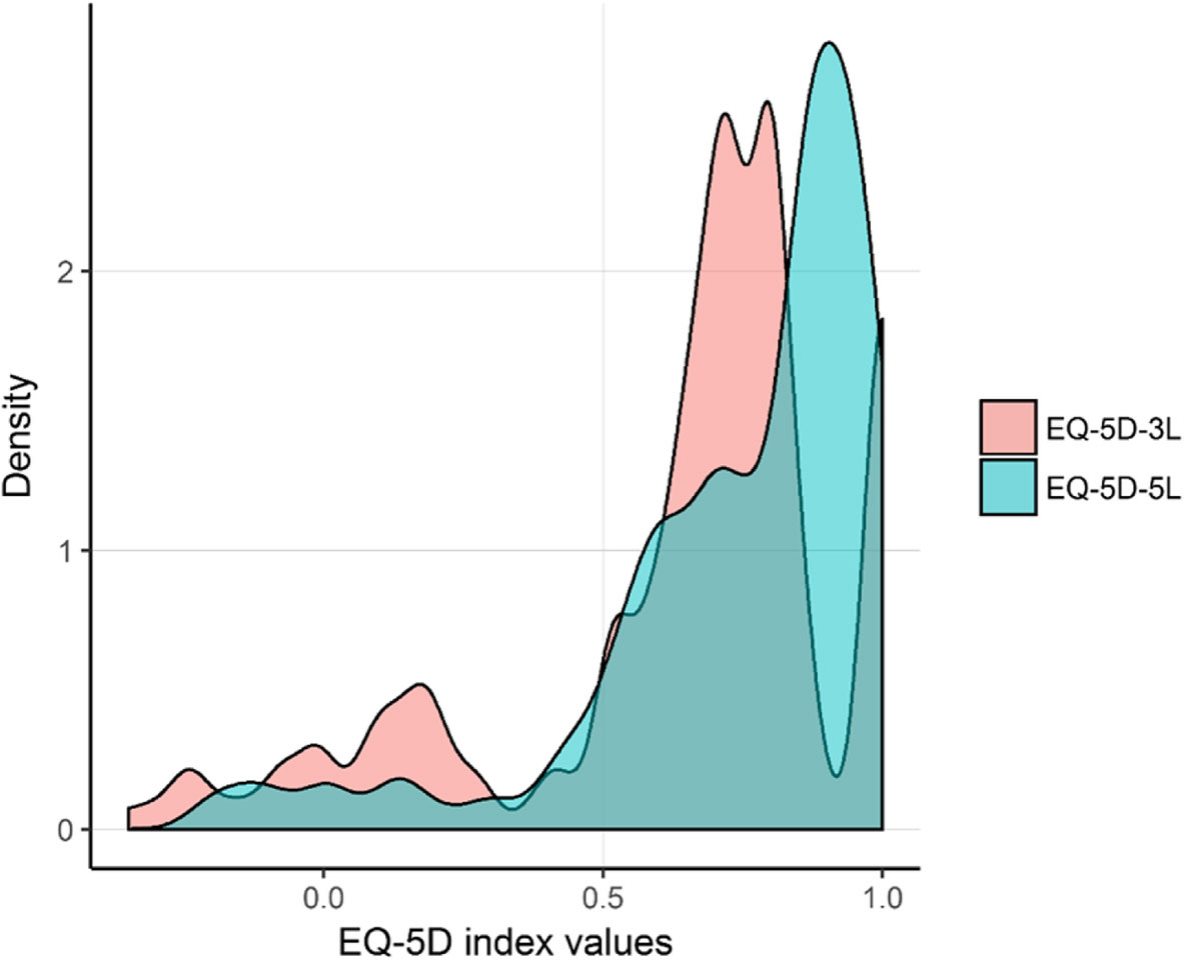
Cumulative percentage of the EQ-5D-3 L and EQ-5D-5 L index scores

The range of index scores was broader in the 3 L than in the 5 L version, especially for negative values (Fig. 1). The lowest index score reported for the 3 L was − 0.349 (state ‘23333’), whereas this was − 0.263 (state ‘45554’) for the 5 L. The most frequently reported health state was ‘11121’ (slight problems in pain/discomfort and no problems in the other dimensions), i.e. 17% in the 3 L and 13% in the 5 L. There were 46 and 90, 3 L and 5 L health states reported in the study, respectively.

### Redistribution from 3 L to 5 L

Of the participants who reported no problem (level 1) for a dimension on the 3 L, most (73–94%) reported the same on the 5 L, while 6–26% switched to slight problems (level 2) on the 5 L as shown in Table 3. The majority of the participants who reported moderate problems (level 2) on the 3 L indicated slight problems (level 2) on the 5 L (44–67%), while 20–28% switched to moderate problems (level 3) and 12–31% shifted to severe problems (level 4) on the 5 L. Most of the participants who indicated confined to bed/unable/extreme problems (level 3) on the 3 L indicated extreme problems (level 5) on the 5 L for the usual activities dimension, whereas most participants who reported extreme problems on 3 L redistributed into severe problems (level 4) for pain/discomfort and anxiety/depression. As for the self-care dimension, these percentages were equal. Redistribution occurred least frequently in the mobility dimension since no participant reported ‘confined to bed’ on the 3 L in that area. The inconsistent responses were ranging from 4% on self-care to 7.6% on the pain/discomfort and anxiety/depression dimensions. An example of such inconsistency was a participant choosing ‘no problems walking’ in 3 L (mobility level 1) and ‘severe problems walking’ in 5 L (mobility level 4).

**Table 3:** Redistribution pattern of response from 3 L to 5 L

Dimension	3 L	5 L	N (%) by 3 L level		Inconsistencies* N (%)
Mobility	1	1	94	(73.08)	11 (5.5)
		2	19	(26.92)	
	2	2	29	(44.74)	
		3	23	(23.68)	
		4	22	(31.58)	
Self-Care	1	1	150	(93.75)	8 (4.0)
		2	10	(6.25)	
	2	2	15	(53.57)	
		3	8	(28.57)	
		4	5	(17.86)	
	3	4	1	(50.00)	
		5	1	(50.00)	
Usual Activities	1	1	117	(89.31)	11 (5.5)
		2	14	(10.69)	
	2	2	22	(45.84)	
		3	13	(27.08)	
		4	13	(27.08)	
	3	4	1	(12.50)	
		5	7	(87.50)	
Pain/Discomfort	1	1	34	(75.55)	15 (7.6)
		2	11	(24.45)	
	2	2	68	(59.65)	
		3	28	(24.56)	
		4	18	(15.79)	
	3	4	15	(65.22)	
		5	8	(34.78)	
Anxiety/Depression	1	1	80	(88.89)	15 (7.6)
		2	10	(11.11)	
	2	2	56	(67.47)	
		3	17	(20.48)	
		4	10	(12.05)	
	3	4	6	(60.00)	
		5	4	(40.00)	

*A consistent response pair was defined as a 3 L response which is at most one level away from the 5 L response (e.g., a participant chose level 1 in 3 L and chose level 2 in 5 L). When the 5 L level was more than 1 level away from the 3 L level (e.g., a participant chose level 1 in 3 L and chose level 3 in 5), this was labelled inconsistent

### Discriminative power

Compared to the 3 L version, the 5 L system had a substantial gain in classification efficiency for each dimension, indicated by higher H′ values of all the dimensions. The J’ values were more similar among the two versions of EQ-5D as shown in Table 4, indicating that the degree of the potential use of the classification system was comparable between the two versions.

**Table 4:** Shannon’s index (H′) and (J’) of 3 L and 5 L

Dimension	H′		J’	
	3 L	5 L	3 L	5 L
Mobility	0.68	1.25	0.43	0.54
Self-care	0.54	0.76	0.34	0.33
Usual activities	0.77	1.10	0.48	0.47
Pain/discomfort	0.94	1.43	0.59	0.62
Anxiety/depression	0.95	1.27	0.60	0.55

### Test-retest reliability

Fifty-three participants (26.8%) completed the instruments twice. By inclusion criterion, all reported no major changes in their health between the first and second data completion point. The weighted kappa of the 5 L dimensions for the 3 L was judged as slightly in agreement for the self-care dimension at 0.14, while the other four dimensions fair agreement existed: mobility at 0.25, usual activities at 0.23, pain/discomfort at 0.25 and anxiety/depression at 0.40. For the 5 L, the pain/discomfort dimension was judged as slightly in agreement at 0.19, while the other four dimensions were in fair agreement: mobility at 0.35, self-care at 0.30, usual activities at 0.37 and anxiety/depression at 0.39. The EQ-VAS ICCs were 0.35 and 0.32 for the 3 L and 5 L respectively. Moreover, the ICCs of the 3 L and 5 L index scores were 0.64 and 0.74 respectively, reflecting a moderate level of reproducibility (Table 5).

**Table 5:** Weighted Kappa and ICC of test-retest

Dimensions	Weighted Kappa	
	EQ-5D-3 L	EQ-5D-5 L
Mobility	0.25	0.35
Self-care	0.14	0.30
Usual activities	0.23	0.37
Pain/Discomfort	0.25	0.19
Anxiety/depression	0.40	0.39
ICC		
VAS scores	0.35	0.32
Index scores	0.64	0.74

## Discussion

We examined some important specific measurement properties of the 3 L and 5 L instruments in Indonesian T2DM outpatients. We found that the 5 L version had a lower ceiling effect, higher discriminative power, and in the majority of the dimensions a higher test-retest reliability coefficient compared to the 3 L*.* The 5 L classification system better represents the variety of patients’ health states, showed by the more health states reported in the 5 L than the 3 L. With regards to the discriminative power, our results showed that 5 L was more discriminative compared to the 3 L, indicated by the gain of the Shannon H′ index from 3 L to 5 L. These results were similar to the findings from across the globe, as reviewed by Buchholz et al. [25]. The J’ index was also in line with the results of the aforementioned study.

The 5 L version showed a lower ceiling effect (health state ‘11111’) than the 3 L at 11 and 15%, respectively. Notably, a previous study [25] suggested that a ceiling effect of 15% and higher should be considered as ‘serious’ (as shown for the 3 L version) while relevantly below 15% is considered small (as shown by the 5 L version). Several studies suggested that other HRQoL instruments have shown lower ceiling effects than the EQ-5D while still strongly correlated with the EQ-5D scores, e.g. the SF-6D [26, 27]. Also, Round suggests to consider other HRQoL measures instead of EQ-5D [28]. However, in several countries, including Indonesia, EQ-5D is the recommended preference-based instrument to measure HRQoL. Therefore, a lower ceiling effect as shown by the 5 L version supports the use of EQ-5D-5 L in Indonesia, especially in patients with T2DM.

Next to better statistical properties, during discussions, also our participants stated that in the 5 L they could more accurately describe their own health state and the severity of T2DM. This is in line with studies in Thailand and Singapore which also stated in both studies that DM severity could be better described in 5 L compared to 3 L [11, 12]. Therefore, our study provides further support to advocate the use of 5 L in clinical, health policy and economic evaluation studies with EQ-5D index score assessments; in our case, notably for Indonesian T2DM outpatients.

Another finding of our research concerns the fact that most participants reported problems on pain/discomfort dimension in the 3 L and 5 L. Notably, the ‘11121’ was the most reported health state by the participants. Four previous studies in Asian populations with T2DM also reported similar findings [12, 29–31]. Also, a multi-country study stated that the Eastern European participants had three times higher mobility and usual activity problems and six times higher self-care problems compared to their Asian counterparts [32].

In this study, the inconsistent responses were ranging from 4% (self-care) to 7.6% (pain/discomfort and anxiety/depression). This was slightly higher than in the studies in China and Singapore at 0.7–1.4% and 2.5–4.1%, respectively. A similar study in Thailand resulted in no inconsistent response at all. It could be argued that higher education level, younger age, and more healthy DM patients (without complications or comorbidities) might play a role in this difference, which indeed seems the case in Thailand study. However, the age distributions and education levels of our participants were overall similar with those in the China and Singapore studies. A possible explanation offered is that the difficulties faced by our elderly participants in completing the 5 L produced these inconsistent responses, although we assisted with explanations. Notably, many elderly participants experienced decreased vision and hearing loss, especially participants in the secondary care facilities. Also, many Indonesian T2DM patients had low levels of education, so an explanation of the HRQoL concept and the EQ-5D instrument was a necessity.

Our study has some limitations which should be considered. First, the participants were recruited from only two locations in Indonesia. Therefore, generalizing the findings nationally should be done with caution. Second, only outpatient participants were recruited for this study. These findings may not be generalizable to inpatients who probably experience more health difficulties: i.e. would report worse health states. Future investigations could include the inpatients to complement the analysis that we provide. Another limitation is that we did not randomize the order of the two versions of the EQ-5D instrument. One could argue that the presentation of 5 L first followed by the 3 L for all participants might produce some bias in the answers of the participants. Our reason was to limit the tendency to not use level 2 and 4 in 5 L [33]. Also, this order was also used in other comparative studies, such as those in Thailand [12], Singapore [11] and one multi-country study Denmark, England, Italy, the Netherlands, Poland, and Scotland [34].

Finally, it is noteworthy that, during our discussions, is seemed that participants with lower education levels and elderly participants preferred the 3 L version, often mentioning that the 3 L version was easier to understand, despite all explanations provided and the flexibility of the 5 L version to more precisely express the health state. Obviously, these patients’ preferences come in as an additional important aspect and warrants further research in this area, inclusive options to even better convey the 5 L version to participants. Finally, further research should focus on other areas in Indonesia beyond our index area of Sulawesi; for example, a similar type of investigation on Java would be worthwhile, with the majority of the Indonesian population living there.

## Conclusion

This study suggests that the 5 L-version of EQ-5D performs better than the 3 L-version in T2DM outpatients in Indonesia. As such, our study supports the use of the 5 L as the preferred HRQoL tool to derive EQ-5D index scores, which ​​are indispensable in pharmacoeconomic analyses and health economic evaluations of interventions in T2DM patients.

## Data Availability

The datasets used and/or analyzed during the current study are available from the corresponding author on reasonable request.

## Abbreviations

3 L: EQ-5D-3 L
5 L: EQ-5D-5 L
DM: Diabetes Mellitus
PHC: Primary Healthcare Centers
T2DM: Type 2 Diabetes Mellitus
